# Fit to Study: Reflections on designing and implementing a large-scale randomized controlled trial in secondary schools

**DOI:** 10.1016/j.tine.2020.100134

**Published:** 2020-09

**Authors:** Catherine Wheatley, Nick Beale, Thomas Wassenaar, Mackenzie Graham, Emma Eldridge, Helen Dawes, Heidi Johansen-Berg

**Affiliations:** a Wellcome Centre for Integrative Neuroimaging, Nuffield Department of Clinical Neurosciences, University of Oxford, John Radcliffe Hospital, Oxford OX3 9DU; b Oxford Institute of Nursing, Midwifery & Allied Health Research, Department of Sport & Health Sciences, Oxford Brookes University, Headington Campus, Oxford OX3 0BP; c Oxford Uehiro Centre for Practical Ethics, Wellcome Centre for Ethics and Humanities, University of Oxford, 6-17 St Ebbes St, Oxford OX1 1PT

**Keywords:** Fit to Study, Randomized controlled trial, Physical activity, Neuroscience, Recruitment, EEthics, Trial management

## Abstract

**Background:**

The randomised controlled trial (RCT) design is increasingly common among studies seeking good-quality evidence to advance educational neuroscience, but conducting RCTs in schools is challenging. Fit to Study, one of six such trials funded by the Education Endowment Foundation and Wellcome Trust, tested an intervention to increase vigorous physical activity during PE lessons on maths attainment among pupils aged 12–13. This review of designing and conducting an RCT in 104 schools is intended as a resource on which researchers might draw for future studies.

**Method:**

We consider intervention design and delivery; recruitment, retention, trial management, data collection and analysis including ethical considerations and working with evaluators.

**Results:**

Teacher training, intervention delivery and data collection during large-scale RCTs require a flexible approach appropriate to educational settings, which in turn entails planning and resources.

**Conclusion:**

Simple interventions, with few outcome measures and minimal missing data, are preferable to more complex designs.

## 1 Introduction

Educational neuroscience has generated much controversy over the past 20 years, so one of the field's key challenges is to provide good-quality evidence showing whether and to what extent laboratory findings can be scaled up and translated into classroom practice [1,2]. A growing number of education studies are using the randomized controlled trial (RCT) design to rigorously test interventions based on novel teaching activities or behavioural strategies informed by science, and to investigate ‘what works’ in schools [3,4]. But conducting an RCT in naturalistic school settings brings considerable practical challenges requiring planning and resources [5,6] and also potential for bias [7]. As a consequence, some study designs now also include implementation and process evaluations to determine what works ‘for whom’ and ‘under what circumstances’ [8].

### 1.1 Fit to Study

In 2014 the Education Endowment Foundation and the Wellcome Trust funded six English projects in which neuroscientists and educators developed and trialled evidence-based interventions for use in the classroom. One of these was Fit to Study (FtS), an RCT that tested whether a programme of vigorous physical activity (VPA) during PE lessons improved brain health and plasticity, and increased maths attainment in Year 8 pupils aged 12–13. The main trial aimed to translate experimental evidence that cardiovascular exercise promotes the development and integration of new blood vessels and neurons in the hippocampus [9] and improves cognitive function [10]. A brain imaging sub-study investigated the underlying neural mechanisms of hypothesized correlations between cardiovascular exercise and cognitive function. Researchers published full details of FtS in the study protocol [11] and the study evaluation report [12].

### 1.2 FtS intervention and primary outcome

PE teachers from intervention schools were trained to deliver a ten-minute warm up at the start of each PE lesson, including four minutes of vigorous physical activity (VPA), and a further three two-minute infusions - short bursts of VPA such as star jumps or running on the spot - per one-hour lesson. Control schools delivered ‘PE as usual’. The intervention ran for a whole school year (2017–2018) and the primary outcome was maths attainment, assessed by the Progress Test in Mathematics (GL Assessment, 2015). Overall, FtS found no evidence that the intervention had an impact on maths outcomes, although the majority of schools said they would recommend FtS as a way of promoting physical activity [12].

### 1.3 FtS trial developers and evaluators

Researchers at the University of Oxford and Oxford Brookes designed the intervention, delivered teacher training, and collected secondary measures of fitness, cognitive function, mental health and VPA during PE [11]. NatCen Social Research, the independent evaluator, set the sample size, collected the primary attainment measure, conducted an implementation and process evaluation, and published the primary results [12].

### 1.4 Aims of this review

EEF, which has funded more than 130 education RCTs, has highlighted key issues to consider when designing and running RCTs in schools, including ensuring interventions are ready for trial; recruiting and retaining schools; calculating sample sizes and ensuring cost-effectiveness; and delivering appropriate testing [5]. Based on our own experiences, this commentary, and associated recommendations (Table 1), aims to provide a further resource on which researchers, evaluators and funding organisations might draw when designing, delivering and measuring the impact of an RCT in the evolving field of educational neuroscience [13]. Some of the issues described are not new - and some are most relevant to physical activity interventions – but failing to consider them could limit progress in this burgeoning field.

**Table 1:** Recommendations for researchers designing and implementing a large trial

Theme	Recommendation
Designing, delivering & measuring interventions	
Design and piloting	• Work with teachers to design a measurable intervention, capable of translating neuroscience theory into teaching practice
Flexible delivery	• Specify how far teachers can deviate from the basic intervention to suit classroom conditions
Fidelity measures	• Specify how fidelity outcomes will account for ‘dose’ variability, e.g a range of compliance cut-offs. Consider pupil-level surveys at baseline and post-intervention and online teacher logs
Blinding control schools	• Prefer a ‘business as usual’ control to an active control
Fostering engagement	• Plan to engage directly with pupils as well as teachers
Recruitment and retention	
Recruitment	• Consider using an independent organisation to manage recruitment in large trials
Retention	• Offer a financial incentive for completing all measures
Workflow planning & trial management	
Scaling up the intervention	• Map social-environmental differences between schools; adapt intervention to suit them or control for variations
Scaling up teacher training	• Schedule training well in advance and support teachers who are cascading training to their departments
Secondary measures	• Prefer fewer, better measurements with less missing data. Make a realistic assessment of resource allocation
Restrictive timelines	• Allocate sufficient resources for measuring and monitoring many schools in a short period
Trial pre-registration	• State hypotheses, sub-group and mediation analyses and describe analysis pipelines prior to data collection
Data collection & analysis	• Consider wider ethical implications of the study aims
Data collection	• Plan time to demonstrate data compliance (GDPR) and to arrange training and permission to collect, store and retrieve pupil data
Data analysis	• Hire a trial statistician or plan additional skills training
Working with teachers	• Establish times to call or email and identify one or two key points of contact per school. Be prepared to accommodate staff absences and unexpected extra-curricular events
Independent evaluation	• Researchers and evaluators must set clear priorities and boundaries for contacting schools and collecting data
Translating results into useful recommendations	• Set effect sizes in the context of the wider education and neuroscience field and consider their practical significance

## 2 Discussion

### 2.1 Designing, delivering and measuring an intervention

#### 2.1.1 Design and piloting

FtS's initial challenge was specifying an intervention that was acceptable and measurable, as well as capable of promoting brain health. The project included an 18-month development phase to design and refine an intervention in consultation with Oxfordshire Sports Partnership and PE teachers. Seven schools (eight recruited; one withdrew) took part in two pilot phases to explore its feasibility and acceptability. The preliminary design was a multi-component approach which aimed to maximize moderate-to-vigorous physical activity (MVPA) in PE lessons using a mix of practical lesson organisation strategies (such as quick changing to increase active lesson time and running small-sized games) and theory-led teaching principles to improve pupils’ self-determined motivation towards PE [14]. This approach was underpinned by evidence that behaviour-change interventions based on psychological theory are more effective than atheoretical approaches [15,16]. The early design also included a Year 8 assembly to explain the purpose of the intervention, and challenging each PE class to record 10,000 min of MVPA in an effort to keep pupils engaged with the task of maximising activity.

But following piloting and consultations with teachers, who recommended a simple, more structured approach, FtS reconfigured the intervention as a set of brief, easy-to-incorporate aerobic exercises intended to directly boost activity and improve cardiovascular fitness and brain health [11]. Unlike a change in teaching style, FtS could then specify the intervention ‘dose’ in terms of frequency (a warm-up and three ‘infusions’), duration (10 minutes per hour of PE) and intensity (vigorous).

A brief, VPA intervention was attractive given competing demands on lesson time and teachers’ capacity to manage additional teaching components. Furthermore, high-intensity activity bursts have been shown to deliver fitness benefits equivalent to longer, lower-intensity workouts [17,18]. We recommend feasibility work with teachers to design and refine an acceptable intervention that is specific, measurable, practical and deliverable both in practice and in theory.

#### 2.1.2 Flexible delivery: one intervention does not fit all

Trial interventions are by definition prescriptive, typically specified by researchers but delivered by teachers, in a real-world environment. A rigid intervention risks undermining teachers’ autonomy and their freedom to adapt an intervention for individual pupils or different settings [1]. But offering too much flexibility can jeopardise intervention fidelity.

FtS therefore specified that teachers could adapt the intervention where necessary by changing the number of infusions if the lesson was significantly longer or shorter than one hour, or by incorporating different (vigorous) exercises to suit the range of sports on the curriculum. Evidence from the process evaluation suggests this proved a popular compromise with teachers, some of whom felt unable to deliver the intervention as prescribed for the full year, for example because students became disengaged or because it interfered with other teaching objectives [12].

*“By tailoring it to our needs and the way we deliver things it has really, really taken off and benefitted the students now.”* (Year 8 teacher, 1059)

*“We're trying to run the curriculum alongside this programme, so it's probably been a bit of a compromise and the best of both worlds.”* (Year 8 teacher, 1101)

Researchers should therefore be prepared for a trade-off between maintaining teacher and pupil engagement and their adherence to a strict intervention delivery method and/or ‘dose’. We recommend specifying in advance how far teachers can deviate from the basic intervention without compromising its impact.

#### 2.1.3 Measuring and monitoring fidelity

FtS was conceived as an efficacy RCT, testing the impact of a full ‘dose’ of an intervention, under ideal and controlled circumstances, with the aim of maximizing the likelihood of detecting any effect. Intervention adaptation, and the move towards an effectiveness-type trial in which real-world effects are measured in non-ideal settings [19] has clear implications for defining and measuring fidelity. FtS's evaluators specified, post-hoc, intervention compliance cut-offs, from 90% to 0% of lessons delivered as specified, and reported the associated complier average causal effects. To map fidelity, researchers triangulated several measures. Asking teachers to keep written day-to-day logs of whether they delivered intervention components was not effective. Completion rates were below 50%, but an online system might potentially have improved engagement. Post-intervention pupil questionnaires asking whether and how often components were delivered was useful, and recommended: similar baseline measurements could have provided a comparison, as could retrospective teacher surveys.

The process evaluation, which consisted of interviews with PE teachers in a sub-set of schools, highlighted practical challenges and teacher preferences that impacted fidelity. For example [12]:

*“There are days where we just can't get it done or we can't implement it in the way that we wished to*.” (Year 8 PE teacher, 1104)

*“Sometimes it could be just that it wasn't feasible inside that lesson to deliver a good or outstanding lesson and have the infusions in there as well.”* (Year 8 PE teacher, 1074)

Researchers also visited schools to observe lessons and measure activity during PE. But the number and geographical spread of schools meant each one could only be visited once per term. Given the large range of sports and physical activities observed, which influence the amount and intensity of overall activity, making unbiased comparisons between intervention and control schools was difficult. The extent to which school-based physical activity interventions are delivered as intended is rarely captured or reported in full [20] and overall FtS also found this aspect challenging.

#### 2.1.4 Blinding control schools

A feature of the RCT design is the ‘double blind’ in which neither participants nor researchers know who is receiving the intervention or the placebo. Blinding schools by developing an active control condition and delivering sham teacher training seemed unnecessarily burdensome. Instead, FtS asked control schools to deliver ‘PE as usual’: this design demonstrates whether the intervention improves outcomes, or at least does no harm, compared to typical practice, although it does not rule out the possibility that simply taking part in any intervention could have delivered similar results. Researchers aimed to prevent control-school teachers absorbing and using the intervention by providing only very general information about its contents prior to randomisation. An unintended consequence of this approach may have been the relatively high attrition rate among intervention schools compared to control once the intervention was revealed (20 intervention schools compared to 11 control schools were lost to follow-up). Nevertheless, we recommend a ‘business as usual’ control to minimize the training burden and to enable comparison with typical current practice.

#### 2.1.5 Fostering intervention engagement

Schools’ enthusiasm for the intervention appeared to wane over the year, despite scope for flexibility. In response, FtS tested initiatives to boost teacher engagement. These included termly school newsletters; an online forum to support the exchange of ideas and experiences between schools; a competition to design and film the most creative infusion; and motivational messages recorded by the Oxford Brookes Chancellor and Olympic oarswoman Dame Katherine Grainger. Interest, although difficult to quantify, appeared limited: anything perceived as an additional burden seems unlikely to gain much traction. Some teachers suggested that engagement effort would be more effective if directed at pupils, for example with a school assembly or promotional materials. Future trials could consider specifying pupil engagement strategies as part of the intervention.

## 3 Recruitment and retention

### 3.1 Recruitment

A second key issue for RCTs in schools is scale. FtS was required to sign up at least 100 schools to adequately power a trial in which whole schools, rather than classes or pupils, were randomised, even though effective recruitment to school-based PA interventions is known to be challenging [6]. The alternative – teaching the intervention to only some students or some classes within schools – brings significant practical problems. The funders therefore commissioned the National Foundation for Economic Research (NfER), an independent research organisation, to recruit state secondary schools with a high proportion of pupils from low-income families. NfER has reported that the importance the education system now places on research is expected to make recruitment easier in the future (NfER, 2018). The collaboration proved a fast and effective method of reaching head teachers from a necessarily wide geographical area.

### 3.2 Retention

The disadvantage of subcontracting recruitment was that developers missed an opportunity to forge relationships with these schools, and to discuss any particular challenges they were facing, at the first point of engagement. This could account for the relatively high rate of attrition in the trial's early stages: of the 106 schools recruited, 11 withdrew before baseline measurements started, citing, for example, staff changes or shortages, work pressure, a behaviourally-challenging year group or forthcoming inspections by Ofsted, the UK's schools’ inspectorate.

**Fig. 1:**
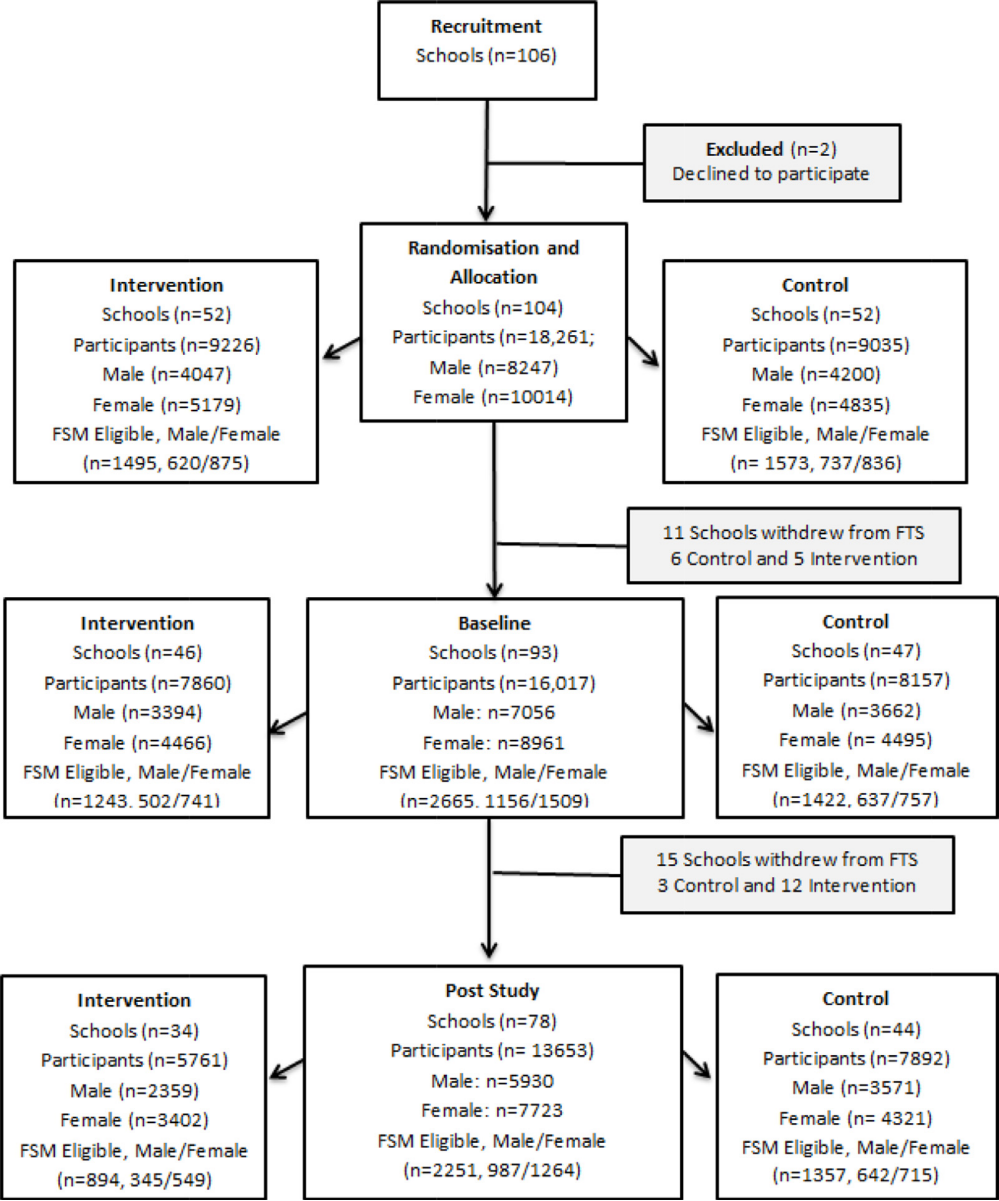
Recruitment and retention through the Fit to Study trial

The number and complexity of outcome measures and evaluations also impacted retention (Fig. 1). Evaluators reported that schools dropped out before and during the primary attainment tests because they clashed with exams and other school activities, and because the logistical difficulties of bringing together pupils for testing were considered burdensome: 44% of pupils selected for maths testing at the start of the trial were not included in their final analysis [12]. To promote retention, FtS offered £500 to PE departments completing all measures over the year, which teachers reported was a positive incentive. Head teachers in EEF trials are expected to sign a Memorandum of Understanding, setting out the school's role and responsibilities, before it is formally recruited [5]: we suggest that teachers tasked with delivering the intervention are also fully informed at this stage.

## 4 Workflow planning and managing a large-scale trial

### 4.1 Scaling up the intervention

What ‘works’ in pilot schools that are culturally and geographically close to researchers’ institutions does not necessarily replicate in a wider context. Likely environmental challenges, including variations in teaching skills, interests and readiness to change, are discussed in the education literature [21]. FtS found, for example, that on scaling up, some teachers reported that intervention training involved too much neuroscience theory and not enough practical teaching suggestions; and that schools with a high proportion of Muslim families did not tolerate VPA well during Ramadan. Scale-up studies, which explicitly examine why and how teachers or schools become willing to adopt and implement new ideas, are complex, time-consuming and relatively uncommon [22,23]. Educationists have suggested that, at a minimum, sampling strategies should include environmental considerations as well as participant characteristics [23]. We recommend mapping social-environmental differences between participating schools where possible, and considering whether to adapt the intervention or, potentially, control for differences.

### 4.2 Scaling up intervention training

Teachers face competing demands on their time: the problems FtS encountered with scheduling intervention training during piloting became more pronounced at scale. In line with good practice, developers conducted pupil-level baseline testing prior to randomisation, which left just weeks before the long holiday to train schools subsequently randomised to the intervention condition. FtS offered online training to teachers unable to attend face-to-face sessions and, when only PE department heads could attend, they were asked to cascade training to their staff. FtS provided these heads with training materials, but in hindsight they should also have been supported to deliver the key points effectively. We recommend scheduling intervention training well in advance. We also suggest asking all schools to set aside an inset day, or other time earmarked for professional development, at the point of recruitment, and then stand down those subsequently allocated to control.

### 4.3 Scaling up work in schools: secondary measures

Extending FtS from seven schools during the pilot phase to 104 schools in the full trial brought significant logistical challenges that were magnified by plans to measure hypothesised mediators of the link between VPA and attainment. Teachers collected fitness data on behalf of the developers by running a Multistage Fitness Test [24] during PE lessons. They were also tasked with delivering computer-based cognitive tasks and an online mental health questionnaire. Although a team of research assistants and PhD students were working full time on the trial, with hindsight this was not sufficient. Problems that could be overcome by spending time in pilot schools became impossible to manage in this way in the larger sample. For example, during piloting, FtS had measured cognitive function on school computers, but installing the necessary browser was difficult because it was blocked by institutional firewalls. Solutions took time, differed from school to school, and required help from school IT staff. In the main trial, participants completed these tasks at home, a pragmatic solution which led, in some cases, to sub-optimal testing conditions and lost data. Alternative solutions might have involved taking dedicated laptop or tablet devices into schools, but this has significant resource implications where large volumes of data are to be collected in parallel over short timescales. With hindsight, the overall testing burden was too great for both teachers and researchers. We recommend making fewer measurements to allow for more reliable measures with less missing data. In line with EEF recommendations [5], we also recommend that funders and developers engage in a realistic analysis of resource allocation, anticipating variations in resource requirements over time, when planning a large-scale trial.

### 4.4 Restrictive timelines

The challenges of managing a large-scale trial were exacerbated by a rigid timetable. FtS ran over a single academic year, which in practice involved working across many geographical locations and socio-economic settings during term time only, to deadlines aligned with school holidays. Off-timetable activities including sports days and school trips, and staff absences and poor weather, reduced the time available for intervention delivery and data collection in some schools relative to others. Overall, teachers were flexible and responded positively to short deadlines. Nevertheless, time constraints, and their impact on intervention delivery affected data collection and fidelity. Allocating resources to allow for simultaneous data collection in all schools, and scheduling multiple measurement days per school, should be considered to reduce missing data and bias.

### 4.5 Trial pre-registration

Pre-registration, which aids transparency and facilitates replication, restricts flexibility in educational settings where day-to-day adaptability is often necessary. But specifying key aspects of a trial, including secondary measures, covariates and fidelity metrics, aids overall planning and offers other researchers a resource when designing RCTs in schools. FtS was pre-registered during data collection and prior to data analysis [11].

## 5 Collecting and analysing data

### 5.1 Ethical considerations

FtS received approval from the University of Oxford's Central University Research Ethics Committee. Head teachers provided informed, written consent on behalf of their schools. The study also used opt-out parental consent, on the basis that opt-in approaches tend to generate smaller samples, less representative of disadvantaged groups [25]. Our experiences during the trial posed a number of ethical questions for future education and neuroscience trials to consider. The issues we outline are not exhaustive, and flow principally from the cluster-RCT (C-RCT) design where schools rather than individuals are assigned to a trial arm: participation affects the interests of all members of the cluster, including teachers and – potentially - parents as well as pupils, although it might not affect them all equally.

*Who is a participant*? Ethical guidelines require that the interests of research participants are protected. It is therefore important that participants in education and neuroscience studies be clearly identified, because not everyone involved in a C-RCT is a participant. According to the Ottawa Statement on the Ethical Design and Conduct of Cluster Randomized Trials, a research participant is anyone whose interests may be affected by a study intervention, or data collection procedure [26]. For example, FtS teachers in the intervention arm attended training, changed their lessons and kept a record of their teaching behaviour. Students received an intervention and had data about them collected. Both groups should be considered participants. Conversely, while teachers and students in the control arm received no intervention, their interests may be affected by lack of access to the intervention. They should also be considered participants.

*Can students avoid participation?* When a ‘gatekeeper’ (such as a head teacher) has the legitimate authority to take decisions on behalf of a cluster (such as a school), they may give *permission* to participate in a trial. This is not a substitute for the (proxy) informed *consent* of individual research participants (e.g., teachers and parents of students). But where a study intervention poses no more than minimal risk —such as that associated with ‘normal school lessons’— a waiver or alteration of consent may be permissible. FtS head teachers consented for all students to take part in the intervention, and to complete secondary measures, as part of normal school lessons, in line with BERA Ethical Guideline for Educational Research [27]. Parents could opt out of data storage on behalf of their children. Some cluster-level interventions may therefore be impossible to avoid, making refusal to participate meaningless. In other kinds of educational neuroscience studies, where participation poses more than a minimal risk, it may be necessary to provide a viable means for students or teachers to decline participation in the intervention.

*Are participation risks and rewards equal within and between clusters?* Clusters may contain a mix of participants, some of whom might be particularly vulnerable to study interventions. Some interventions are ideally suited to active or high-achieving classes or students: for example the FtS intervention suited students who were confident performing VPA with their peers, while others refused to participate. By contrast, novel learning activities might be particularly unsuitable for students with specific learning difficulties, for example. When assessing their study, researchers and research ethics committees should account for potential differences in the benefits and harms of a study intervention for different participants.

### 5.2 Data collection

Under data protection regulations, FtS researchers required training and certification to access moderator variables such as participants’ previous exam scores and other information from the UK National Pupil Database. Researchers are recommended to plan in advance to arrange authorization to collect and store pupil data, and to retrieve sensitive data stored by a third party.

### 5.3 Data analysis

Modelling C-RCT data is complex and requires advanced techniques. Multilevel approaches, which account for the fact that pupils in the same school tend to be more similar to one another than to those in other schools, are becoming more common thanks to improvements in computational power and statistical software [28], but they are typically beyond the scope of standard statistics modules. Pre-planning all secondary outcomes, sub-group and mediation analyses is recommended, as is considering requirements for a trial statistician or additional skills training.

*Interdisciplinary collaboration: scientists working with schools.* UK teachers are under strain [29], so it is encouraging to note their enthusiasm for taking part in education and neuroscience research in addition to existing commitments. PE departments appeared keen to work with researchers, not least to find evidence supporting PE's role in the curriculum [12]:

*“One of the main motivating factors, I suppose, was to highlight the importance of PE potentially in wider school provision.”* (Year 8 PE teacher, 1017)

Nevertheless, researchers and teachers have different priorities, timetables and working environments, which sometimes caused practical issues. Many of these problems are common and well-documented [30,31]. Classroom teachers and lab-based researchers have fundamentally different working styles: we recommend arranging in advance the best times to call, email or otherwise contact one another, and to identify one or two key points of contact in each school. Researchers should also be prepared to accommodate staff absences and extra-curricular events when planning site visits. Protocols for sending and receiving confidential pupil-level data should be agreed with schools in advance.

## 6 Independent evaluation

EEF/Wellcome commissioned NatCen to undertake the trial's independent implementation and process evaluation. Study roles and responsibilities were therefore divided between the University of Oxford and Oxford Brookes (the developers) and NatCen Social Research (the evaluators). We support independent scrutiny of the research process. We welcome process evaluations that address the ‘for whom’ and ‘under what circumstances’ of education trials, and give teachers the opportunity to provide feedback on education and neuroscience studies. We also note the potential for tension in a working arrangement that involves a unidirectional critique of procedures throughout the trial process. An unintended consequence of the arrangement was two different groups, with different sets of priorities, were both in contact with schools. This caused confusion among PE departments – with, for example, different contact details and information leaflets - and frustrated researchers who were trying to build strong relationships with teachers. Oxford researchers and the evaluation team agreed to time frames within which only one group would approach schools: this appeared to lessen confusion but further reduced available time for testing. Some teachers suggested that the burden of intervention training, lesson monitoring, data collection and process evaluation interviews - all within just a few weeks - was considerable, and this might have contributed to the attrition rate. Regular, constructive communication between academic researchers and evaluators is essential, and the partners should set clear priorities and boundaries for contacting schools and collecting data.

## 7 Translating results into useful recommendations

Controlling for Key Stage 2 maths results (at Year 6), the intervention's standardised effect size, measured by Hedges’ g, was -0.008 (CI -0.06, 0.05) [12]. This was less than the average effect size across all EEF-funded trials of 0.1 standard deviations (as of 2017) and considerably smaller than 0.24, the average standard effect size of the most promising EEF trials with results deemed strong enough to justify re-grant funding [5]. Sub-group analyses by sex and free school meal status had similar results. Many well-designed education interventions fail to detect an effect [32]. Furthermore, multi-school trials with over 250 participants report effect sizes around half the size of those derived from smaller studies, and RCTs report significantly smaller effect sizes than matched experiments [33]. We suggest that studies set effect sizes in the context of the wider field and consider what practical significance, if any, an observed difference might have. One possibility would be to compare these results against observed effect sizes for similar interventions. FtS was set in the context of per pupil cost, a key metric for policymakers and head-teachers. Over three years, the estimated per pupil cost of delivering FtS, assuming face-to-face teacher training, was just £4.80. For comparison, the EEF suggests that interventions costing less than £80 per pupil per 0.1 standard deviation are considered ‘very good’ value for money Evaluators might consider calculating the ratio of cost to effect size and comparing this figure to the results of other intervention studies.

## 8 Conclusions

Designing and delivering RCTs that produce good-quality evidence to advance educational neuroscience is challenging. Researchers, in collaboration with teachers, should plan to deliver an RCT design as fully as possible given the available resources, which include staff to recruit, train, test and monitor a potentially large number of schools, and teachers’ capacity to assist with testing and deliver adapted lessons over a period of weeks or months. Schools’ needs should be kept central to the research with early planning to improve communication and implementation. Given the practical issues involved in measuring ‘what works’ in school settings, consider in advance how to define and measure fidelity and effect sizes, and how to capture the ‘for whom’ and ‘under what circumstances’ aspects of the trial. The experience of working with PE teachers during FtS suggests a brief, simple, flexible intervention is more sustainable over an academic year than a complex, multi-component approach. A successful trial is one where these issues are considered and their outcomes published, regardless of any effect that may or not be found.

## Declaration of Competing Interest

The authors declare no conflict of interest other than the funding sources described.
